# Transgenerational effects of the genocide against the Tutsi in Rwanda: A post-traumatic stress disorder symptom domain analysis

**DOI:** 10.12688/aasopenres.12848.1

**Published:** 2018-04-18

**Authors:** Susan Rudahindwa, Leon Mutesa, Eugene Rutembesa, Jean Mutabaruka, Annie Qu, Derek E. Wildman, Stefan Jansen, Monica Uddin

**Affiliations:** 1 Carl R. Woese Institute for Genomic Biology, University of Illinois at Urbana Campaign, Champaign, IL, USA; 2 Department of Psychology, University of Illinois at Urbana Campaign, Champaign, IL, USA; 3 Center for Human Genetics, University Teaching Hospital of Kigali, Kigali, Rwanda; 4 Department of Clinical Psychology, University of Rwanda, Huye, Rwanda; 5 Department of Statistics, University of Illinois at Urbana Champaign, Champaign, IL, USA; 6 Department of Molecular and Integrative Physiology, University of Illinois at Urbana Campaign, Champaign, IL, USA; 7 Center for Mental Health, College of Medicine and Health Sciences, University of Rwanda, Kigali, Rwanda; 8 Directorate of Research and Innovation, College of Medicine and Health Sciences, University of Rwanda, Kigali, Rwanda

**Keywords:** Prenatal Stress, Mental Health, Tutsi, Glucocorticoid Receptor, Confirmatory Factor Analysis

## Abstract

**Background:** A number of studies have investigated transgenerational effects of parental post-traumatic stress disorder (PTSD) and its repercussions for offspring. Few studies however, have looked at this issue in the African context.

**Methods:** The present study addresses this gap, utilizing confirmatory factor analysis (CFA), to investigate symptom severity within the three Diagnostic and Statistical Manual of Mental Disorders IV (DSM-IV) PTSD symptom domains in mothers exposed to the genocide against the Tutsi in Rwanda (n=25) and offspring (n=25), and an ethnically matched control group of mothers (n=25) and offspring (n=25) who were outside of Rwanda during the genocide. All mothers were pregnant during the time of the genocide with the offspring included in the study. Missing data were excluded from the analyses.

**Results:** We found that among the three symptom domains of PTSD, the re-experiencing symptom domain loaded most strongly onto PTSD among mothers directly exposed to the genocide (Beta = 0.95). In offspring of exposed mothers, however, the three symptom domains of PTSD yielded almost equal loading values (Beta range = 0.84-0.86). Conversely, among non-exposed mothers and their offspring, the hyperarousal symptom domain of PTSD loaded most strongly onto PTSD (Beta = 1.00, Beta = 0.94, respectively). As a secondary analysis, we also explored the relation between DNA methylation in the glucocorticoid receptor ( *NR3C1)* locus, an important stress modulating gene, and individual PTSD symptom domains, finding a strong association between DNA methylation and re-experiencing among genocide-exposed mothers that exceeded any other observed associations by approximately two-fold.

**Conclusions**: This is the first report, to our knowledge, of a symptom-based analysis of transgenerational transmission of PTSD in Africa. These findings can be leveraged to inform further mechanistic and treatment research for PTSD.

## Introduction

The 1994 genocide perpetrated against Tutsi in Rwanda is one of the most horrific events in recent history responsible for roughly one million deaths during a short 3-month period. The genocide was a result of tension between the two artificially divided ethnic groups of Rwanda: Hutu and Tutsi. Hutu extremists, alongside coerced and willing civilians, carried out a mass genocide against the minority ethnic Tutsi of Rwanda ^1– 3^. Throughout the region, high rates of post-traumatic stress disorder (PTSD) and other psychological and physical disorders have been observed following the 1994 genocide perpetrated against Tutsi ^4– 7^. A recent countrywide study concluded that 79.41% of Rwandan individuals (age >16) experienced one or more traumatic events, including threats of death, bodily injury to a person or member of his family, being a witness of killing of a family member or another member of the community, and rape ^4^. Women were to a higher extent exposed to traumatic events (83.6%) than men (73.4%) ^8^. More than two decades later, the long-term impact of the genocide is evident in the prevalence of PTSD – more than 26% among Rwandans (age>16) – and even higher rates (41%) among women survivors ^4, 7^ Work published in 2013 analyzing PTSD prevalence among Rwandan widows, prisoners of war, and their descendants reported that high exposure to war and genocide was one of the greatest predictors of PTSD ^6^. 

Further study has also uncovered the transgenerational effects of PTSD among genocide-exposed mothers and their offspring ^9, 10^. Perroud and his colleagues found that offspring of genocide-exposed mothers had higher rates of PTS and depressive symptom severity than offspring born from non-exposed mothers ^9^. These findings are consistent with research reported on holocaust survivors and their adult offspring, which also depicted higher rates of PTSD among offspring of holocaust survivors compared to those of non-exposed parents ^11^.

While PTSD or PTSD severity is often the primary phenotype of interest in studies of traumatic stress, numerous studies have implemented confirmatory factor analysis (CFA) models of PTSD as a means of testing a hypothesized model that may characterize in more detail potential indicators of PTSD (e.g. 12– 14). The purpose of such analysis is to test which set of symptom domains collectively best describe PTSD, by finding the best fit model. Following the Diagnostic and Statistical Manual of Mental Disorders IV (DSM-IV) ^15^, the first symptom domain is a persisting re-experiencing of the traumatic event, including flashbacks, intrusive thoughts, and nightmares. The second symptom domain is a persistent avoidance of trauma-associated stimuli and a numbing of general responsiveness, which includes feelings of detachment from others, effortful avoidance of thought, feelings, and activities that arouse recollections of the trauma, and a restricted range of affect. The third symptom domain is a persistence of hyperarousal symptoms, which include insomnia, hypervigilance, and exaggerated startle response ^15^.

Traditionally, a CFA is used to test how well a hypothesized model holds and how well the model fits the observed data ^12, 13^. In contrast, the purpose of the present study is to apply a CFA analysis in order to determine which symptom domain has the greatest loading on PTSD in the context of the 1994 genocide perpetrated against the Tutsi in Rwanda. The ultimate aim of this study is to elucidate potential transgenerational differences in how exposure to the 1994 genocide perpetrated against Tutsi affects PTS symptom severity domains. More specifically, CFA was used in this study to extract domain loading values on PTSD in order to assess which of the three symptom domains serves as the strongest indicator of PTSD in mothers exposed and pregnant during genocide and their offspring from this pregnancy, as well as in a comparison group of non-exposed mothers (living outside of Rwanda) pregnant in that same period and their offspring from this pregnancy. From here on, we will be referring to these groups as exposed mothers and offspring and non-exposed mothers and offspring. Our primary interest was in the potential differences in loading values observed in exposed mothers compared to their offspring and in non-exposed mothers compared to their offspring, respectively.

As a secondary analysis, we tested how strongly these symptom domains were associated with epigenetic modifications. Epigenetic modifications can be understood as overlying genetic modifications occurring on a gene in a manner that can potentially alter gene expression ^16^. These epigenetic modifications are modulated by personal experiences and can be inherited in some cases ^17, 18^. Thus, adversities—particularly early life adversities--experienced throughout one’s life can alter functional properties of a gene through changed epigenetics and, in some instances, may also be observed on the offspring’s epigenome ^19^. Complementing the animal work, which can more directly demonstrate transgenerational epigenetic effects from a mechanistic perspective, a handful of human-based studies has reported stress- and trauma-associated epigenetic patterns in one generation that appear to persist into a subsequent generation, in particular among genes that help to regulate the body’s response to stress ^9, 20– 22^. In particular, Perroud *et al*. ^9^ found that both mothers exposed to the 1994 genocide perpetrated against Tutsi and their offspring had a positive relationship between PTSD symptom severity and blood-derived DNA methylation of the glucocorticoid receptor gene ( *NR3C1)*. However, the extent to which *NR3C1* DNA methylation associates with particular PTSD symptoms domains remains unknown. Therefore, following our CFA analysis of PTSD symptom domains, we conducted a secondary analysis to assess which symptom domain, as outlined above, has the strongest association with DNA methylation in *NR3C1* for both mothers and offspring.

## Methods

### Participants and measures

This study utilized PTSD Checklist (PCL-17) responses of 50 Tutsi women and their offspring, obtained from previous study, as described by Perroud *et al.* ^9^. Data were collected from 2011–2012. The PCL-17 questionnaire used in this study was administered by trained psychologists. For the case group, 25 ethnic Tutsi widows exposed to the genocide, and pregnant during the time, were recruited from a Tutsi genocide Widows’ Association and/or psychiatric ambulatory consultations, along with their 25 offspring who were *in utero* during this period (data were missing for two offspring from the exposed group). Participation for mothers in the study was following DSM-IV criterion A, which states that an individual must have witnessed or been threatened by death or serious injury that evoked intense fear, helplessness, or horror ^23^. The control group consisted of 25 women of Tutsi ethnicity who pregnant during, but not exposed to the 1994 genocide perpetrated against Tutsi, along with their 25 offspring from this pregnancy. For the 25 non-exposed women, those with a known exposure to traumatic experiences and history of a psychiatric disorder were excluded from the study. All participants provided informed consent.

### DNA methylation measures

DNA methylation measurements of *NR3C1* used in this study were previously obtained by Perroud *et al.* ^9^. Briefly, DNA was first extracted, bisulfite converted, and amplified via PCR. After pyrosequencing, percent mean methylation values at 10 discrete CpG sites within the exon 1 _F_ *NR3C1* promoter region were determined for both exposed and non-exposed mothers as well as their offspring; average values across the 10 CpG sites were also determined and used as the primary variable in our secondary analyses reported here.

### Statistical analysis

**Figure 1.**
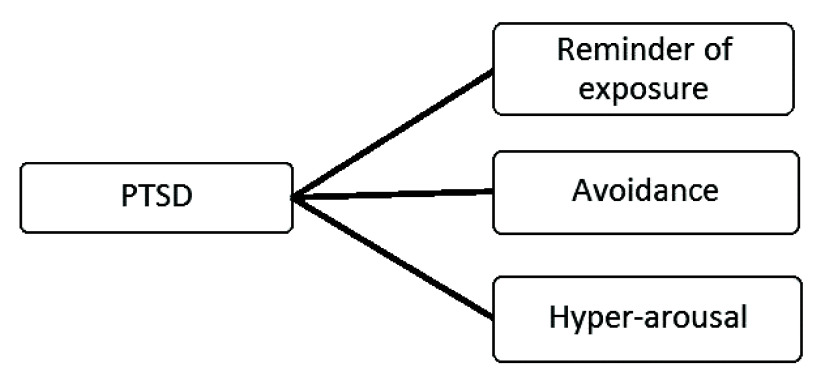
Post-traumatic stress disorder (PTSD) model for confirmatory factor analysis (CFA).

To study the relationship between PTSD symptom domains and genocide exposure a confirmatory factor analysis (CFA) model was used testing the individual loading values of each symptom domain onto PTSD ( Figure 1). This model is based on Buckley *et al*. ^12^ who used a similar approach to study PTSD symptom domains. The model, depicted in Figure 1, was assessed four times: one model each for the exposed mothers and offspring; a third model for non-exposed mothers; and a fourth model for their offspring.

We used analysis of moment structures (AMOS) ^24^. PTSD was set as the latent variable and the three symptom domains were set as indicators of the latent variable. Beta values derived from the CFA analysis indicate loading values of each symptom domain onto the latent variable PTSD. Results were then used to compare symptom domains among the four different groups (exposed mother and offspring, non-exposed mother and offspring).

 For the secondary analyses of DNA methylation in relation to PTSD symptom domains, we implemented for each of the four groups a Bayesian model to construct a matrix that would give us covariant values of how strongly each symptom domain is related to average DNA methylation of the exon 1 _F_ *NR3C1* promoter region as reported in 9. This analysis was coded in RStudio version 1.1.423 ^25^ by following the Bayesian equation with normal Wishart distribution ^26^. Missing data were excluded from the analyses.

## Results

### Primary CFA analyses of PTSD symptom domains

**Figure 2.**
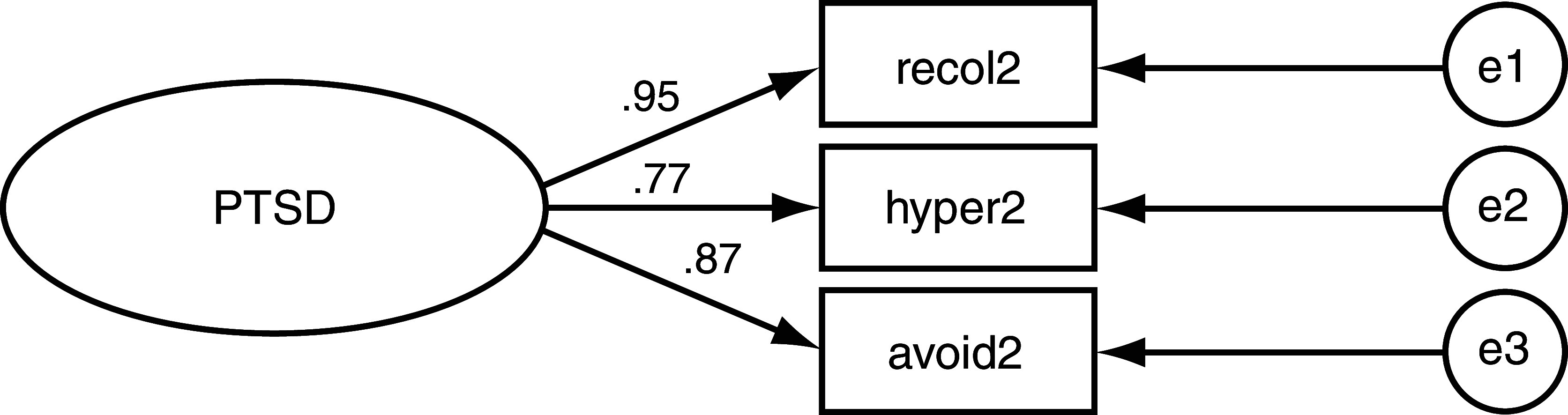
Post-traumatic stress disorder (PTSD) Structural Equation Model for exposed mothers. Loading values indicated on leftmost arrows. Recol2 = Re-experiencing. Hyper2 = Hyper arousal. Avoid2 = Avoidance/Numbing. e = error. e1 =.001 e2 = .002 e3 =.003.

**Figure 3.**
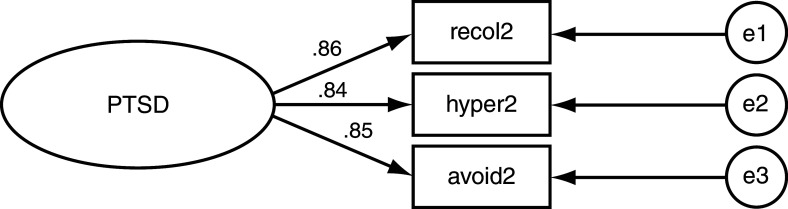
Post-traumatic stress disorder (PTSD) Structural Equation Model for offspring of exposed mothers. Loading values indicated on leftmost arrows. Recol2 = Re-experiencing. Hyper2 = Hyper arousal. Avoid2 = Avoidance/Numbing. e = error e1 = .004 e2 = .003 e3 =.003.

**Figure 4.**
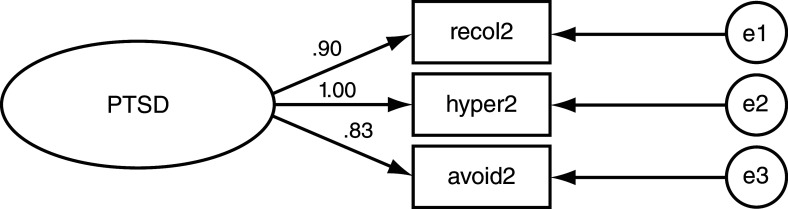
Post-traumatic stress disorder (PTSD) Structural Equation Model for non-exposed mothers. Loading values indicated on leftmost arrows. Recol2 = Re-experiencing. Hyper2 = Hyper arousal. Avoid2 = Avoidance/Numbing. e = error e1 = .004 e2 = .003 e3 =.004.

**Figure 5.**
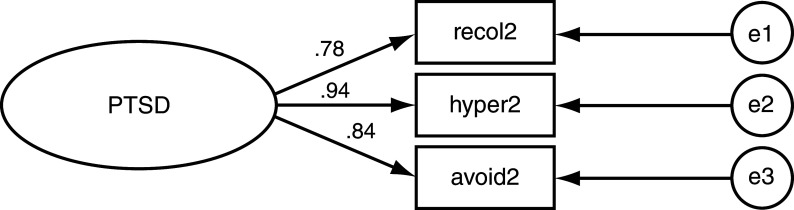
Post-traumatic stress disorder (PTSD) Structural Equation Model for offspring of non-exposed mothers. Loading values indicated on leftmost arrows. Recol2 = Re-experiencing. Hyper2 = Hyper arousal. Avoid2 = Avoidance/Numbing. e = error e1 = .006 e2 = .003 e3 =.004.

CFA showed that among the genocide-exposed mothers, re-experiencing symptoms had the highest loading value onto PTSD (Beta = 0.95; Table 1; Figure 2). In contrast, the symptoms categorized under hyperarousal had the lowest loading value (Beta = 0.77). Symptoms categorized as avoidance/numbing had the second largest loading value (Beta = 0.87). In offspring of genocide-exposed mothers, re-experiencing symptoms, hyperarousal symptoms, and avoidance/numbing symptoms had almost equal loading values, respectively, on PTSD (Beta = 0.86, Beta = 0.84, Beta = 0.85; Table 1; Figure 3). Among non-exposed mothers, hyperarousal symptoms had the highest loading value on PTSD (Beta = 1.00; Table 2; Figure 4) followed by re-experiencing (Beta = 0.90) and avoidance/numbing (Beta = 0.83). Similarly, in offspring of non-exposed mothers, hyperarousal symptoms loaded on PTSD most strongly (Beta = 0.94; Table 2; Figure 5); but avoidance/numbing symptoms had a higher loading value than re-experiencing symptoms (Beta=0.84 vs. 0.78, respectively).

**Table 1.** Symptom domain loading values for exposed mothers and offspring.

Latent Factor	Indicator	Beta coefficients for exposed mothers	Beta coefficients for exposed offspring
PTSD	Re-experiencing	0.95	0.86
PTSD	Hyperarousal	0.77	0.84
PTSD	Avoidance/Numbing	0.87	0.85

Table 1. Factor Loadings. Latent Factor = PTSD, Indicator = symptom domains, Beta = symptom severity by loading value. PTSD - post-traumatic stress disorder.

**Table 2.** Symptom domain loading values for non-exposed mothers and offspring.

Latent Factor	Indicator	Beta coefficients for non-exposed mothers	Beta coefficients for non-exposed offspring
PTSD	Re-experiencing	0.90	0.78
PTSD	Hyperarousal	1.00	0.94
PTSD	Avoidance/Numbing	0.83	0.84

Table 2. Factor Loadings. Latent Factor = PTSD, Indicator = symptom domains, Beta = symptom severity by loading value. PTSD - post-traumatic stress disorder

### Secondary analyses of NR3C1 DNA methylation in relation to PTSD symptom domains

Among mothers exposed to the genocide, the re-experiencing symptom domain had the strongest relationship with *NR3C1* DNA methylation levels (Covariance = 0.20; Table 3), with the hyperarousal symptom domain and avoidance/numbing symptom domain showing equivalently strong relationships with *NR3C1* methylation (Covariance = 0.13 for both domains). Among offspring of exposed mothers, the hyperarousal symptom domain and avoidance/numbing symptom domain also had an equal relationship with methylation of *NR3C1* (Covariance = 0.10 for both domains; Table 3); however, in contrast to the pattern observed in the exposed mothers, the re-experiencing symptom domain had the weakest relationship with methylation of *NR3C1* (Covariance = 0.06).

**Table 3.** Covariance table for exposed mothers and offspring.

	*NR3C1* Methylation covariance for exposed mothers	*NR3C1* Methylation covariance for offspring of exposed mothers
Re-experiencing	0.20	0.06
Hyperarousal	0.13	0.10
Avoidance/Numbing	0.13	0.10

Among non-exposed mothers the avoidance/numbing symptom domain had the strongest relationship with DNA methylation in *NR3C1* (Covariance = 0.10; Table 4), followed by the re-experiencing (Covariance = 0.08), and hyperarousal (Covariance = 0.06) symptom domains. Like non-exposed mothers, the covariance matrix for offspring of non-exposed mothers showed that the avoidance/numbing symptom domain had the strongest relationship with DNA methylation in *NR3C1*(Covariance = 0.11; Table 4), followed closely by the re-experiencing symptom domain (Covariance = 0.10) and, lastly, the hyper arousal symptom domain (Covariance = 0.07).

**Table 4.** Covariance table for non-exposed mothers and offspring.

	*NR3C1* Methylation covariance for non- exposed mothers	*NR3C1* Methylation covariance for offspring of non-exposed mothers
Re-experiencing	0.08	0.10
Hyperarousal	0.06	0.07
Avoidance/Numbing	0.10	0.11

## Discussion

Our primary CFA analyses showed that among the genocide exposed mothers, symptoms categorized under the domain of re-experiencing had the highest loading value onto PTSD. These findings suggest that exposed mothers experienced more nightmares, intrusive thoughts, and flashbacks—hallmark symptoms of this domain within the PTSD diagnosis ^15^. Intuitively, we assume that the high loading value associated with the re-experiencing symptom domain seen in exposed mothers is a consequence of their direct witnessing of the trauma: since the mothers directly witnessed the trauma, they are the ones with the ability to recall the event. In contrast, their children, who were *in utero* during this time, did not bear witness to the atrocities of the genocide. Therefore, it is not surprising that their children would experience a different level or set of symptom severities. Perhaps more interestingly, offspring of genocide-exposed mothers did not experience one symptom domain more severely than another, suggesting that, at least at the level of symptoms, there is a lack of transgenerational symmetry in the genocide-exposed group.

In contrast, among ethnic Tutsis not exposed directly to the genocide, the hyperarousal symptom domain loaded most strongly onto PTSD among both the mothers and the children. One possibility is that, within this group, being a member of a minority ethnic group that was the target of genocide promoted vigilant behaviors characteristic of this symptom domain; in other words, these individuals may have been prone to express hyperarousal symptoms as a response to being potential targets of genocide, even though that threat may not have been imminent as these individuals were outside of Rwanda at the time. This could be interpreted as an extreme version of social identity threat theory, in which individuals of minority backgrounds experience heightened vigilance to social environmental cues as they seek information about whether their identity may be a source of potential mistreatment (reviewed in 27).

Results from our secondary analyses showed that DNA methylation on the exon 1 _F_ promoter region of *NR3C1* had the strongest relationship with the re-experiencing symptom domain of PTSD for genocide exposed mothers. This follows the same pattern as the results from our CFA analysis, which indicated that the re-experiencing symptom domain loaded most strongly onto PTSD in genocide exposed mothers. However, unlike their genocide exposed mothers, genocide-exposed offspring did not show associations with DNA methylation that paralleled the primary CFA results (i.e. the three PTSD symptom domains were not equally related to the DNA methylation status of *NR3C1*). Rather, the hyperarousal and avoidance/numbing symptom domains showed the strongest relationship with methylation of the *NR3C1* exon 1 _F_ promoter region in this group, with equal covariance values; in contrast, the re-experiencing symptom domain of PTSD had the weakest relationship with methylation of *NR3C1* for offspring of genocide exposed mothers. Of note, in the Perroud *et al*. study ^9^, the offspring of genocide-exposed mothers showed the highest levels of DNA methylation in *NR3C1*, which were nearly 50% higher than that observed in their mothers. Interestingly, in our CFA-based approach reported here, the mothers demonstrated an association with *NR3C1* DNA methylation in relation to the re-experiencing symptom domain that exceeded any observed association between *NR3C1* DNA methylation and symptom domains in their offspring (or in any other group) by twofold or greater. This suggests that, while *in utero* exposure to genocide may have a pronounced effect on overall DNA methylation at this locus, the more subtle relationship between *NR3C1* methylation and PTSD symptom domains may be more evident within generations.

A number of limitations should be considered in evaluating the results of the current study. Firstly, the psychopathological assessments and accompanying blood sample collection of offspring included in the study occurred more than a decade following their *in utero* exposure to the genocide. More specifically, offspring of genocide exposed and non-exposed mothers were already of adult age when recruited into the original study ^9^, thus results from the present study may be influenced by post-natal factors such as: parental rearing methods, constant exposure to maternal PTSD symptoms, and other stressors acquired throughout the offspring’s lifetime, therefore making it difficult to differentiate pre-natal environmental effects from post-natal factors. Therefore, we cannot attribute the results of this study solely to in-utero exposure to the genocide. Secondly, our secondary analyses utilized group mean DNA methylation values, drawing from previous work ^9^, instead of individual DNA methylation percentages of each participant, which could add variability to the reported results. To conduct our study using the mean methylation values taken from the previous study, we simulated data that would fall within the expected distribution given the mean and standard deviation of each group. Covariance values indicating the relationship strength between DNA methylation of *NR3C1* and each PTSD symptom domain were then presented and discussed in terms of their ranking from highest to lowest. Future studies should extend the approach presented in this study to create a linear regression model of DNA methylation of *NR3C1* and PTSD symptom domains given actual percent DNA methylation values for each study participant. Lastly, our study only explored the relationship of DNA methylation on *NR3C1* and PTSD symptom domains. Future studies should explore the relationship of PTSD symptom domains on DNA methylation in other genes related to PTSD.

Despite these limitations, the present study highlights the relationship between specific symptom domains and PTSD within a transgenerational context and, secondarily, with epigenetic modifications in a stress-sensitive gene. The novelty in this study is its transgenerational illustration of PTSD symptom severity domains and their possible relationship to DNA methylation of *NR3C1* in the African context. In particular, its goal was to investigate the nature of PTSD symptom severity domains as it relates to genocide exposure against the Tutsi in Rwanda. We found that, among those with direct exposure to the Tutsi genocide in Rwanda, the re-experiencing symptom domain showed the strongest loading onto PTSD. Future studies should investigate whether similar relations hold for individual symptom domains in other trauma exposed populations, as well as identify potential biomarkers of the individual symptom domains for other trauma exposed populations.

## Conclusion

Examination of transgenerational effects associated with traumatic event exposure is an active area of research. Results from the present study not only lend another perspective on the relative severity of PTSD symptom domains experienced by trauma exposed individuals, in particular within an African context; they also illustrate how *in utero* exposure to trauma associates with offspring symptom severities and how such symptoms are, in turn, related to epigenetic modifications in stress related genes. Future work in our ongoing study will examine transgenerational effects of genocide exposure on mental health in a larger sample of study participants and will broaden epigenetic analyses to include a broader suite of genes involved with stress regulation.

## Data availability

The data underlying this study is available from OSF: http://doi.org/10.17605/OSF.IO/P94TA ^28^.

Data are available under the terms of the Creative Commons Zero "No rights reserved" data waiver (CC0 1.0 Public domain dedication).

The authors responsible for the data described have agreed to make them publicly accessible.

## References

[1] Schaal S Elbert T : Ten years after the genocide: trauma confrontation and posttraumatic stress in Rwandan adolescents. *J Trauma Stress.* 2006;19(1):95–105. 10.1002/jts.20104 16568463

[2] Dyregrov A Gupta L Gjestad R : Trauma exposure and psychological reactions to genocide among Rwandan children. *J Trauma Stress.* 2000;13(1):3–21. 10.1023/A:1007759112499 10761171

[3] Mutabaruka J Séjourné N Bui E : Traumatic grief and traumatic stress in survivors 12 years after the genocide in Rwanda. *Stress Health.* 2012;28(4):289–96. 10.1002/smi.1429 22282057

[4] Schaal S Weierstall R Dusingizemungu JP : Mental health 15 years after the killings in Rwanda: imprisoned perpetrators of the genocide against the Tutsi versus a community sample of survivors. *J Trauma Stress.* 2012;25(4):446–53. 10.1002/jts.21728 22865747

[5] Rubanzana W Hedt-Gauthier BL Ntaganira J : Exposure to genocide and risk of suicide in Rwanda: a population-based case-control study. *J Epidemiol Community Health.* 2015;69(2):117–22. 10.1136/jech-2014-204307 25488977 PMC4316837

[6] Rieder H Elbert T : Rwanda - lasting imprints of a genocide: trauma, mental health and psychosocial conditions in survivors, former prisoners and their children. *Confl Health.* 2013;7(1):6. 10.1186/1752-1505-7-6 23531331 PMC3620568

[7] Munyandamutsa N Mahoro Nkubamugisha P Gex-Fabry M : Mental and physical health in Rwanda 14 years after the genocide. *Soc Psychiatry Psychiatr Epidemiol.* 2012;47(11):1753–61. 10.1007/s00127-012-0494-9 22402589

[8] Rugema L Mogren I Ntaganira J : Traumatic episodes experienced during the genocide period in Rwanda influence life circumstances in young men and women 17 years later. *BMC Public Health.* 2013;13:1235. 10.1186/1471-2458-13-1235 24373422 PMC3880849

[9] Perroud N Rutembesa E Paoloni-Giacobino A : The Tutsi genocide and transgenerational transmission of maternal stress: epigenetics and biology of the HPA axis. *World J Biol Psychiatry.* 2014;15(4):334–45. 10.3109/15622975.2013.866693 24690014

[10] Roth M Neuner F Elbert T : Transgenerational consequences of PTSD: risk factors for the mental health of children whose mothers have been exposed to the Rwandan genocide. *Int J Ment Health Syst.* 2014;8(12). 10.1186/1752-4458-8-12 24690436 PMC3978019

[11] Yehuda R Bell A Bierer LM : Maternal, not paternal, PTSD is related to increased risk for PTSD in offspring of Holocaust survivors. *J Psychiatr Res.* 2008;42(13):1104–11. 10.1016/j.jpsychires.2008.01.002 18281061 PMC2612639

[12] Buckley TC Blanchard EB Hickling EJ : A confirmatory factor analysis of posttraumatic stress symptoms. *Behav Res Ther.* 1998;36(11):1091–9. 10.1016/S0005-7967(98)00076-X 9737061

[13] Andrews L Joseph S Shevlin M : Confirmatory factor analysis of posttraumatic stress symptoms in emergency personnel: An examination of seven alternative mdoels. *Pers Individ Dif.* 2006;41(2):213–24. 10.1016/j.paid.2005.11.034

[14] Ismail K Everitt B Blatchley N : Is there a Gulf War syndrome? *Lancet.* 1999;353(9148):179–82. 10.1016/S0140-6736(98)11339-9 9923872

[15] American Psychiatric Association, American Psychiatric Association: Task Force on DSM-IV. Diagnostic and statistical manual of mental disorders: DSM-IV. 4th ed. Washington, DC: American Psychiatric Association;1994;xxvii:886. Reference Source

[16] Paul B Tollefsbol TO : Outline of Epigenetics.In Peedicayil J, Grayson DR, Avramopoulos D, editors. *Epigenetics in Psychiatry*. San Diego, CA: Elsevier;2014;27–44. 10.1016/B978-0-12-417114-5.00002-4

[17] Gapp K Soldado-Magraner S Alvarez-Sánchez M : Early life stress in fathers improves behavioural flexibility in their offspring. *Nat Commun.* 2014;5:5466. 10.1038/ncomms6466 25405779

[18] Gapp K Jawaid A Sarkies P : Implication of sperm RNAs in transgenerational inheritance of the effects of early trauma in mice. *Nat Neurosci.* 2014;17(5):667–9. 10.1038/nn.3695 24728267 PMC4333222

[19] Bale TL : Epigenetic and transgenerational reprogramming of brain development. *Nat Rev Neurosci.* 2015;16(6):332–44. 10.1038/nrn3818 25921815 PMC7064155

[20] Mulligan CJ D'Errico NC Stees J : Methylation changes at *NR3C1* in newborns associate with maternal prenatal stress exposure and newborn birth weight. *Epigenetics.* 2012;7(8):853–7. 10.4161/epi.21180 22810058 PMC3427280

[21] Yehuda R Daskalakis NP Bierer LM : Holocaust Exposure Induced Intergenerational Effects on *FKBP5* Methylation. *Biol Psychiatry.* 2016;80(5):372–80. 10.1016/j.biopsych.2015.08.005 26410355

[22] Kertes DA Kamin HS Hughes DA : Prenatal Maternal Stress Predicts Methylation of Genes Regulating the Hypothalamic-Pituitary-Adrenocortical System in Mothers and Newborns in the Democratic Republic of Congo. *Child Dev.* 2016;87(1):61–72. 10.1111/cdev.12487 26822443 PMC4733886

[23] Association AP : American Psychiatric Association’s Diagnostic and Statistical Manual of Mental Disorders (DSM-IV).1994;1232.

[24] Arbuckle JL : Amos for windows. Analysis of Moment Structures.Chicago.1999.

[25] RStudio Team: RStudio: Integrated Development Environment for R. RStudio, Inc. Boston, MA.2016.

[26] Penny W : Bayesian Inference for the Multivariate Normal.Wellcome Trust Center for Neuroimaging: University College, London.2014. Reference Source

[27] Emerson KT Murphy MC : Identity threat at work: how social identity threat and situational cues contribute to racial and ethnic disparities in the workplace. *Cultur Divers Ethnic Minor Psychol.* 2014;20(4):508–20. 10.1037/a0035403 25133411

[28] Mutesa L : PTSD Symptom Domain Data. *Open Science Framework.* 2018. 10.17605/OSF.IO/P94TA

