# Transgenerational effects of the genocide against the Tutsi in Rwanda: A post-traumatic stress disorder symptom domain analysis

**DOI:** 10.12688/aasopenres.12848.2

**Published:** 2020-01-14

**Authors:** Susan Rudahindwa, Leon Mutesa, Eugene Rutembesa, Jean Mutabaruka, Annie Qu, Derek E. Wildman, Stefan Jansen, Monica Uddin

**Affiliations:** 1 Carl R. Woese Institute for Genomic Biology, University of Illinois at Urbana Campaign, Champaign, IL, USA; 2 Department of Psychology, University of Illinois at Urbana Campaign, Champaign, IL, USA; 3 Genomics Program, College of Public Health, University of South Florida, Tampa, FL, USA; 4 Center for Human Genetics, University Teaching Hospital of Kigali, Kigali, Rwanda; 5 Center for Human Genetics, College of Medicine and Health Sciences, University of Rwanda, Kigali, Rwanda; 6 Department of Clinical Psychology, University of Rwanda, Huye, Rwanda; 7 Department of Statistics, University of Illinois at Urbana Champaign, Champaign, IL, USA; 8 Department of Statistics, University of California, Irvine, Irvine, CA, USA; 9 Department of Molecular and Integrative Physiology, University of Illinois at Urbana Campaign, Champaign, IL, USA; 10 Center for Mental Health, College of Medicine and Health Sciences, University of Rwanda, Kigali, Rwanda; 11 Directorate of Research and Innovation, College of Medicine and Health Sciences, University of Rwanda, Kigali, Rwanda

**Keywords:** Prenatal Stress, Mental Health, Tutsi, Glucocorticoid Receptor

## Abstract

**Background:** A number of studies have investigated transgenerational effects of parental post-traumatic stress disorder (PTSD) and its repercussions for offspring. Few studies however, have looked at this issue in the African context.

**Methods:** The present study addresses this gap by utilizing a Pearson correlation matrix to investigate symptom severity within the three Diagnostic and Statistical Manual of Mental Disorders IV (DSM-IV) PTSD symptom domains in mothers exposed to the genocide against the Tutsi in Rwanda (n=25) and offspring (n=25), and an ethnically matched set of controls (n=50) who were outside of Rwanda during the 1994 genocide. All mothers were pregnant with the offspring included in the study during the time of the genocide.

**Results:** Total PTS score was significantly (p<0.01) correlated with each of the three symptom domains at various strengths in both cases and controls. No significant differences in association of total PTS score and PTSD symptom domains were observed between exposed mothers and offspring, suggesting that each symptom domain contributed equivalently to both exposed mothers and offspring distress. In contrast, the re-experiencing symptom domain showed a significant difference in correlation to overall PTS score in non-exposed mothers compared to their offspring (p<0.05), with mothers showing a significantly higher correlation. Furthermore, the correlation between avoidance/numbing symptoms to overall PTS was significantly different (p≤0.01) across exposed and non-exposed mothers. As a secondary analysis, we explored the relationship between DNA methylation in the glucocorticoid receptor (NR3C1) locus, an important stress modulating gene, and PTSD symptom domains, finding an association between DNA methylation and re-experiencing among genocide-exposed mothers that exceeded any other observed associations by approximately two-fold.

**Conclusions**: This is the first report, to our knowledge, of a symptom-based analysis of transgenerational transmission of PTSD in sub-Saharan Africa. These findings can be leveraged to inform further mechanistic and treatment research for PTSD.

## Introduction

The 1994 genocide perpetrated against Tutsi in Rwanda is one of the most horrific events in recent history responsible for roughly one million deaths during a short 3-month period. The genocide was a result of tension between the two artificially divided ethnic groups of Rwanda: Hutu and Tutsi. Hutu extremists, alongside coerced and willing civilians, carried out a mass genocide against the minority ethnic Tutsi of Rwanda ^1– 3^. Throughout the region, high rates of post-traumatic stress disorder (PTSD) and other psychological and physical disorders have been observed following the 1994 genocide perpetrated against Tutsi ^4– 7^. A recent countrywide study concluded that 79.41% of Rwandan individuals (age >16) experienced one or more traumatic events, including threats of death, bodily injury to a person or member of his family, being a witness of killing of a family member or another member of the community, and rape ^4^. Women were exposed to traumatic events to a higher extent than men (83.6% vs. 73.4%) ^8^. More than two decades later, the long-term impact of the genocide is evident in the prevalence of PTSD – more than 26% among Rwandans (age>16) – and even higher rates (41%) among women survivors ^4, 7^ Work published in 2013 analyzing PTSD prevalence among Rwandan widows, prisoners of war, and their descendants reported that high exposure to war and genocide was one of the greatest predictors of PTSD ^6^.

Further study has also uncovered the transgenerational effects of PTSD among genocide-exposed mothers and their offspring ^9, 10^. Perroud and his colleagues found that offspring of genocide-exposed mothers had higher rates of PTS and depressive symptom severity than offspring born from non-exposed mothers ^9^. These findings are consistent with research reported on holocaust survivors and their adult offspring, which also depicted higher rates of PTSD among offspring of holocaust survivors compared to those of ethnically matched non-exposed parents ^11^.

Overall PTSD or PTSD severity is often the primary phenotype of interest in studies of traumatic stress. In this study, we chose instead to explore how individual symptom domains of PTSD is manifested in Tutsi survivors of the genocide and their offspring alongside an ethnically matched control group. Following the Diagnostic and Statistical Manual of Mental Disorders IV (DSM-IV) ^12^, the first symptom domain is a persistent re-experiencing of the traumatic event, including flashbacks, intrusive thoughts, and nightmares. The second symptom domain is a persistent avoidance of trauma-associated stimuli and a numbing of general responsiveness, which includes feelings of detachment from others, effortful avoidance of thought, feelings, and activities that arouse recollections of the trauma, and a restricted range of affect. The third symptom domain is a persistence of hyperarousal symptoms, which include insomnia, hypervigilance, and exaggerated startle response ^12^.

The ultimate aim of this study is to elucidate potential transgenerational differences in how exposure to the 1994 genocide perpetrated against Tutsi affects PTSD symptom severity domains. More specifically, a symptom by symptom matrix was constructed, results summarized in Table 1 and Table 2, in order to assess which of the three symptom domains is most strongly associated with overall PTS (post-traumatic stress) severity score in mothers exposed and pregnant during the genocide and their offspring from this pregnancy, as well as in a comparison group of non-exposed mothers (living outside of Rwanda) pregnant in that same period and their offspring from this pregnancy. From here on, we will be referring to these groups as exposed mothers and offspring and non-exposed mothers and offspring. Our primary interest was in the potential difference(s) in association of total PTS score and PTSD symptom domains in exposed mothers compared to their offspring and in non-exposed mothers compared to their offspring, respectively. We also tested the difference in association of total PTS score and PTSD symptom domains within generations across exposure groups, that is, between exposed and non-exposed mothers and between exposed and non-exposed offspring.

**Table 1.** Overall PTSD and PTS symptom domain correlation for exposed mothers and offspring.

Symptom Domain	Correlation with overall PTSD symptom severity score, exposed mothers	Correlation with overall PTSD symptom severity score, offspring of exposed mothers
Re-experiencing	.92 **	.89 **
Hyperarousal	.92 **	.90 **
Avoidance/Numbing	.89 **	.91 **

**. Correlation is significant at or below the 0.01 level (2-tailed).

**Table 2.** Overall PTSD and PTS symptom domain correlation for non-exposed mothers and offspring.

Symptom Domain	Correlation with overall PTSD symptom severity score, non- exposed mothers	Correlation with overall PTSD symptom severity score, offspring of non-exposed mothers
Re-experiencing	.95 **	.81 **
Hyperarousal	.89 **	.87 **
Avoidance/Numbing	.97 **	.94 **

**. Correlation is significant at or below the 0.01 level (2-tailed).

As a secondary analysis, we tested how strongly these symptom domains were associated with epigenetic modifications. Epigenetic modifications can be understood as overlying DNA sequences in a manner that can potentially alter gene expression ^13^. These epigenetic modifications are modulated by personal experiences and can be inherited in some cases ^14, 15^. Thus, adversities—particularly early life adversities--experienced throughout one’s life can alter functional properties of a gene through changed epigenetics and, in some instances, may also be observed on the offspring’s epigenome ^16^. Complementing the animal work, which can more directly demonstrate transgenerational epigenetic effects from a mechanistic perspective, a handful of human-based studies has reported stress- and trauma-associated epigenetic patterns in one generation that appear to persist into a subsequent generation, in particular among genes that help to regulate the body’s response to stress ^9, 17– 19^. In particular, Perroud *et al*. ^9^ found that both mothers exposed to the 1994 genocide perpetrated against Tutsi and their offspring had a positive relationship between PTSD symptom severity and blood-derived DNA methylation of the glucocorticoid receptor gene ( *NR3C1)*. However, the extent to which *NR3C1* DNA methylation associates with particular PTSD symptoms domains remains unknown. Therefore, following our primary analysis of PTSD symptom domains, we conducted a secondary analysis to assess which symptom domain, as outlined above, has the largest association with DNA methylation in *NR3C1* for both mothers and offspring.

## Methods

### Participants and measures

This study utilized PTSD Checklist (PCL-17) responses of 50 Tutsi women and their offspring, obtained from previous study, as described by Perroud *et al.* ^9^. Data were collected from 2011–2012. The PCL-17 questionnaire used in this study was administered by trained psychologists. For the case group, 25 ethnic Tutsi widows exposed to the genocide, and pregnant during the time, were recruited from a Tutsi genocide Widows’ Association and/or psychiatric ambulatory consultations, along with their 25 offspring who were *in utero* during this period (data were missing for two offspring from the exposed group). Participation for mothers in the study was following DSM-IV criterion A, which states that an individual must have witnessed or been threatened by death or serious injury that evoked intense fear, helplessness, or horror ^20^. The control group consisted of 25 women of Tutsi ethnicity who were pregnant during, but not exposed to the 1994 genocide perpetrated against Tutsi, along with their 25 offspring from this pregnancy. All participants provided informed consent.

### DNA methylation measures

DNA methylation measurements of *NR3C1* used in this study were previously obtained by Perroud *et al.* ^9^. Briefly, DNA was first extracted, bisulfite converted, and amplified via PCR. After pyrosequencing, percent mean methylation values at 10 discrete CpG sites within the exon 1 _F_ *NR3C1* promoter region were determined for both exposed and non-exposed mothers as well as their offspring; average values across the 10 CpG sites were also determined and used as the primary variable in our secondary analyses reported here.

### Statistical analysis

To study the relationship between PTSD symptom domains and genocide exposure we first log transformed our data and visualized it using a scatter plot. After visually confirming a linear relationship, via scatter plot, between PTSD symptom domains and overall PTS we constructed a Pearson correlation matrix to test the strength and significance of each symptom domain with overall PTS symptom severity score within each of the four groups (exposed mothers, exposed offspring, non-exposed mothers, non-exposed offspring). The Pearson correlation matrix was conducted in SPSS (IBM Corp, Armonk, NY) and a matrix was constructed for each of the four groups. Following construction of the Pearson correlation matrix, we then transformed our Pearson correlation coefficients (r-scores) into z-scores to test for significant differences in correlations with overall PTS symptom severity score; all possible pairwise comparisons between symptom domains were tested. For example, for each group we tested if the correlation coefficient of hyperarousal symptoms to overall PTS severity was significantly different than the correlation coefficient between avoidance/numbing and overall PTS severity. Here Z-score-based differences in correlation were tested within and across our 4 groups. The transformation and comparison of our correlation coefficients was done in Rstudio version 1.1.423 ^21^ using the cocor package.

For the secondary analyses of DNA methylation in relation to PTSD symptom domains, we implemented for each of the four groups a Bayesian model to construct a matrix that would give us covariant values of how each symptom domain is related to average DNA methylation of the exon 1 _F_ *NR3C1* promoter region as reported in 9. This analysis was coded in RStudio version 1.1.423 ^21^ by following the Bayesian equation with normal Wishart distribution ^22^. Participants with missing data were excluded from the analyses.

## Results

### Primary analyses of PTSD symptom domains

Table 1 shows results for the correlation of each of the three PTSD symptom domains with overall PTS severity scores in genocide-exposed mothers and genocide-exposed offspring. Table 2 shows results for the correlation of each of the three PTSD symptom domains with overall PTS severity scores in non-exposed mothers and their offspring. All four groups showed significant (p<0.01) correlations between each of the three symptom domains and overall PTS score.

Despite this shared pattern, Z -score analyses within each of the four groups showed significant differences in the strength of association between select symptom domains and overall PTS severity score. Specifically, non-exposed mothers showed a significant (p≤0.001) difference in correlation between hyperarousal and avoidance/numbing symptom domains in relation to overall PTS severity score, with avoidance/numbing symptoms showing a significantly higher correlation ( Table 3). In addition, in offspring of non-exposed mothers, avoidance and numbing symptoms showed a significant difference (p≤0.01) in correlation to overall PTS severity score compared to re-experiencing symptoms, with avoidance/numbing symptoms again showing a significantly higher correlation ( Table 3).

**Table 3.** Z-score test of pairwise difference in symptom clusters’ association with overall PTSD severity score, within groups.

	Rxp/Avoid		Rxp/Hyper		Hyper/avoid	
	**Z score**	**p value**	**Z score**	**p value**	**Z score**	**p value**
**Mx**	0.82	0.40	0.02	0.98	0.88	0.38
**Kx**	-0.50	0.62	-0.11	0.91	-0.39	0.69
**M**	-1.65	0.10	1.76	0.08	-3.27	.001 **
**K**	-2.57	0.01 *	-0.87	0.39	-1.74	0.08

Table 3. Dunn and Clark Z-test. Mx = exposed mothers, Kx = offspring of exposed mothers, M = non-exposed mothers, K = offspring of non-exposed mothers. *. *Correlation is significantly different at or below the 0.05 level (2-tailed).* **. *Correlation is significantly different at or below the 0.01 level (2-tailed).*

Furthermore, analyses of specific PTSD symptom domains between groups showed that the correlation between re-experiencing symptoms and overall PTS score was significantly different (p≤0.01) when comparing non-exposed mothers and their offspring, with mothers showing a significantly higher correlation ( Table 4). Lastly, the correlation coefficient of avoidance and numbing symptoms to overall PTS score was significantly different (p<0.05) between non-exposed mothers compared to exposed mothers, with non-exposed mothers showing a significantly higher correlation ( Table 4). No other significant differences in Z-score tests of PTSD symptom domains were observed between groups.

**Table 4.** Z-score test of pairwise difference in symptom clusters’ association with overall PTSD severity score, among groups.

	Rxp		Avoid		Hyper	
	**Z score**	**p value**	**Z score**	**p value**	**Z score**	**p value**
Mx/Kx	0.69	0.49	-0.43	0.67	0.52	0.60
M/K	2.21	0.03 *	1.42	0.15	0.31	0.76
*Mx/M*	-0.65	0.52	-2.46	0.01 *	0.51	0.61
*Kx/K*	0.89	0.37	-0.65	0.51	0.47	0.64

Table 4. Dunn and Clark Z-test. Mx = exposed mothers, Kx = offspring of exposed mothers, M = non-exposed mothers, K = offspring of non-exposed mothers. *. Correlation is significantly different at or below the 0.05 level (2-tailed).

### Secondary analyses of NR3C1 DNA methylation in relation to PTSD symptom domains

Among mothers exposed to the genocide, of the three symptom domains, the re-experiencing symptom domain had the largest relationship with *NR3C1* DNA methylation levels ( Table 5), with the hyperarousal symptom domain and avoidance/numbing symptom domain showing equivalent relationships with *NR3C1* methylation ( Table 5). Among offspring of exposed mothers, the hyperarousal symptom domain and avoidance/numbing symptom domain also had an equal relationship with methylation of *NR3C1* ( Table 5); however, in contrast to the pattern observed in the exposed mothers, the re-experiencing symptom domain had the smallest relationship with methylation of *NR3C1* ( Table 5).

**Table 5.** Covariance table for exposed mothers and offspring.

Symptom Domain	*NR3C1* Methylation covariance for exposed mothers	*NR3C1* Methylation covariance for offspring of exposed mothers
Re-experiencing	0.20	0.06
Avoidance/Numbing	0.13	0.10
Hyperarousal	0.13	0.10

Among non-exposed mothers the hyperarousal symptom domain had the largest relationship with DNA methylation in *NR3C1*, followed by the re-experiencing and avoidance/numbing symptom domains ( Table 6). Like non-exposed mothers, the covariance matrix for offspring of non-exposed mothers showed that the hyperarousal symptom domain had the largest relationship with DNA methylation in *NR3C1*, followed closely by the re-experiencing symptom domain and, lastly, the avoidance/numbing symptom domain ( Table 6).

**Table 6.** Covariance table for non-exposed mothers and offspring.

Symptom Domain	*NR3C1* Methylation covariance for non- exposed mothers	*NR3C1* Methylation covariance for offspring of non- exposed mothers
Re-experiencing	0.08	0.10
Avoidance/Numbing	0.06	0.07
Hyperarousal	0.10	0.11

## Discussion

In this study we used a symptom-based approach to investigate PTS transmission in women survivors of the 1994 genocide against the Tutsi in Rwanda and their offspring, as well as an ethnically matched group of women and their offspring living outside of Rwanda during the genocide. Addressing our primary interest, we found that although each PTSD symptom domain correlated significantly with overall PTS severity scores at various r-scores, the pairwise associations did not significantly differ between exposed mothers and their offspring. That is, although symptoms classified under the re-experiencing and hyperarousal domain had a higher correlation value to overall PTS scores in exposed mothers, this correlation did not differ from their offspring. These results suggest that PTS in offspring of mothers exposed to the 1994 genocide against the Tutsi were driven by the same set of symptoms as their mothers. In contrast, the association of re-experiencing symptoms to overall PTS was significantly higher in non-exposed mothers compared to their offspring. These results indicate that non-exposed mothers experienced relatively more flashbacks, intrusive thoughts, and nightmares compared to their offspring. Furthermore, that these experiences had a greater influence on PTS in non-exposed mothers compared to their offspring. However, all other symptom associations with overall PTS scores did not significantly differ across non-exposed mothers and offspring. This indicates that exposure to extreme trauma (i.e. genocide) produces PTSD symptoms across domains that are experienced similarly/equivalently between generations and that, in this context, domain-specific differences of loading onto PTS severity score are not relevant to the intergenerational transmission of PTS.

In addition to the intergenerational results, within-group analyses of PTSD symptom domains revealed additional noteworthy findings. Specifically, in non-exposed mothers, the association of avoidance and numbing symptoms to overall PTS score was significantly higher when compared to the association of hyperarousal symptoms to overall PTS score, indicating that non-exposed mothers had greater avoidance and numbing symptoms than hyperarousal symptoms. Similarly, offspring of non-exposed mothers experienced more avoidance and numbing symptoms than re-experiencing symptoms. This suggests that avoidance and numbing symptoms were among the most prevalent symptoms in both non-exposed mothers and their offspring.

Additionally, we were interested in how PTS symptoms may differ within generations but between exposure groups. What we found was that exposed mothers had fewer symptoms of avoidance and numbing compared to non-exposed mothers. In contrast, no observed differences were found between offspring of exposed mothers and offspring of non-exposed mothers. These results may be contextualized by prior studies of other genocide-survivors and matched control groups, as well as the cultural context of Rwandans themselves within and outside of the country during the 1994 genocide. For example, a case-control study of successful aging in offspring of holocaust survivors and an ethnically matched control group, described that almost half of the parents not exposed to the holocaust reported a sudden loss of a loved one as a traumatic event ^23^. This is important to note because although the comparison group was not directly exposed to the holocaust, the tragedy still had some degree of impact on their psyche. Thus, due to the magnitude of casualties during the 1994 genocide in Rwanda, it is very possible that in the current study the PTS symptoms observed in non-exposed mothers may be due to traumatic events related to losing a loved one and other close ties in their homeland. However, as described in 24, Rwandans living in Canada explained that silence was the most culturally appropriate response to the genocide. Thus, it is possible that those living abroad during the 1994 genocide followed the same cultural norm and relied more on avoidant behaviors to cope. One possibility is that not being able to talk freely about the genocide may have led to the prominence of avoidance and numbing symptoms in Tutsi women living abroad (Rutembesa, pers. comm). Furthermore, offspring of non-exposed mothers may then have inadvertently learned to rely more on avoidant behaviors as a coping mechanism from their mothers. In contrast, exposed mothers were primarily recruited from survivor association groups which means they belonged to a group where they could speak openly around other women survivors who experienced similar traumatic experiences. We suggest that this key difference may be the reason why avoidance and numbing symptoms were less prevalent in Tutsi women living in Rwanda as compared to Tutsi women living outside of Rwanda.

Results from our secondary analyses showed that, among the three symptom domains, DNA methylation on the exon 1 _F_ promoter region of *NR3C1* had the largest relationship with the re-experiencing symptom domain of PTSD for genocide exposed mothers. However, unlike their genocide exposed mothers, the hyperarousal and avoidance/numbing symptom domain showed the largest relationship with methylation of the *NR3C1* exon 1 _F_ promoter region in this group, with equal covariance values; in contrast, the re-experiencing symptom domain of PTSD had the smallest relationship with methylation of *NR3C1* for offspring of genocide exposed mothers. Of note, in the Perroud *et al*. study ^9^, the offspring of genocide-exposed mothers showed the highest levels of DNA methylation in *NR3C1*, which were nearly 50% higher than that observed in their mothers. Interestingly, the mothers demonstrated an association with *NR3C1* DNA methylation in relation to the re-experiencing symptom domain that exceeded any observed association between *NR3C1* DNA methylation and symptom domains in their offspring (or in any other group) by twofold or greater. This suggests that, while *in utero* exposure to genocide may have a pronounced effect on overall DNA methylation at this locus, the relationship between *NR3C1* methylation and PTSD symptom domains may be more evident within generations. We have no clear explanation for why this can be the case, so more study is needed to understand this observation.

A number of limitations should be considered in evaluating the results of the current study. Firstly, the psychopathological assessments and accompanying blood sample collection of offspring included in the study occurred almost two decades following their *in-utero* exposure to the genocide. More specifically, offspring of genocide exposed and non-exposed mothers were already of adult age when recruited into the original study ^9^, thus results from the present study may be influenced by post-natal factors such as: parental rearing methods, constant exposure to maternal PTS symptoms, and other stressors acquired throughout the offspring’s lifetime, therefore making it difficult to differentiate pre-natal environmental effects from post-natal factors. Moreover, we did not formally assess secondary traumatization, which has been shown to mediate the relationship between maternal PTS and offspring PTS in Holocaust survivors ^11, 25, 26^. Recent preliminary findings on a sample of Tutsi survivors and their adult offspring also reported an association between parental PTSD and complex PTSD (CPTSD) with offspring secondary traumatization ^27^. Due to these limitations we cannot link the results of this study solely to in-utero exposure to the genocide.

Secondly, our secondary analyses utilized group mean DNA methylation values, drawing from previous work ^9^, instead of individual DNA methylation percentages of each participant, which could add variability, as we cannot account for real effects of the data distribution, to the reported results. To conduct our study using the mean methylation values taken from the previous study, we simulated data that would fall within the expected distribution given the mean and standard deviation of each group. Covariance values indicating the relationship strength between DNA methylation of *NR3C1* and each PTSD symptom domain were then presented and discussed in terms of their ranking from largest to smallest. Future studies should extend the approach presented in this study to create a linear regression model of DNA methylation of *NR3C1* and PTSD symptom domains given actual percent DNA methylation values for each study participant. Lastly, our study only explored the relationship of DNA methylation on *NR3C1* and PTSD symptom domains. Future studies should explore the relationship of PTSD symptom domains on DNA methylation in other genes related to PTSD.

Despite these limitations, the present study highlights the relationship between specific symptom domains and PTSD within a transgenerational context and, secondarily, with epigenetic modifications in a stress-sensitive gene. The novelty in this study is its transgenerational illustration of PTSD symptom severity domains and their possible relationship to DNA methylation of *NR3C1* in the African context. In particular, its goal was to investigate the nature of PTSD symptom severity domains as it relates to genocide exposure against the Tutsi in Rwanda. We found that, each PTSD symptom domain contributed to PTS severity equivalently in Tutsi mothers exposed to the 1994 genocide and their offspring. This is in contrast to Tutsi mothers living outside of Rwanda who experienced greater re-experiencing symptoms compared to their offspring, and both non-exposed mothers and their offspring showed higher symptoms of avoidance and numbing symptoms as compared to one other PTSD symptom domain. Furthermore, avoidance and numbing symptoms were significantly less expressed in exposed mothers compared to non-exposed mothers.

Future studies should conduct similar symptom-based PTSD analyses utilizing the DSM-5’s definition and clustering of PTSD, which expands and clusters avoidance and numbing symptoms separately ^28^. Cross cultural examinations of PTSD have shown that differential clustering of symptoms result in different rates of individual PTSD symptoms (reviewed in 29). Therefore, a symptom-based analysis of PTSD utilizing the DSM-5’s clustering of PTSD may capture differences in PTSD symptoms that we were unable to capture with the three-factor clustering of PTSD in the DSM-IV ^12^, as assessed in this study. Future studies should also investigate whether similar relations hold for individual symptom domains in other trauma exposed populations, as well as identify potential biomarkers of the individual symptom domains for trauma exposed populations.

## Conclusion

Examination of transgenerational effects associated with traumatic event exposure is an active area of research. The primary results of this study add to the growing evidence of PTSD prevalence in offspring of mothers exposed to genocidal traumas ^9, 11^, and does so within an African context. However, the current study uncovers an additional level of evidence beyond overall PTSD severity, showing that observed levels of distress in exposed mothers and offspring are driven by the same symptoms, whereas non-exposed mothers and offspring differ by at least one PTSD symptom domain. Future work in our ongoing study will examine transgenerational effects of genocide exposure on mental health in a larger sample of study participants and will broaden epigenetic analyses to include a broader suite of genes involved with stress regulation.

## Data availability

The raw (non-transformed) data underlying this study is available from OSF: http://doi.org/10.17605/OSF.IO/P94TA ^30^.

Data are available under the terms of the Creative Commons Zero “No rights reserved” data waiver (CC0 1.0 Public domain dedication).

The authors responsible for the data described have agreed to make them publicly accessible.

## References

[1] Schaal S Elbert T : Ten years after the genocide: trauma confrontation and posttraumatic stress in Rwandan adolescents. *J Trauma Stress.* 2006;19(1):95–105. 10.1002/jts.20104 16568463

[2] Dyregrov A Gupta L Gjestad R : Trauma exposure and psychological reactions to genocide among Rwandan children. *J Trauma Stress.* 2000;13(1):3–21. 10.1023/A:1007759112499 10761171

[3] Mutabaruka J Séjourné N Bui E : Traumatic grief and traumatic stress in survivors 12 years after the genocide in Rwanda. *Stress Health.* 2012;28(4):289–96. 10.1002/smi.1429 22282057

[4] Schaal S Weierstall R Dusingizemungu JP : Mental health 15 years after the killings in Rwanda: imprisoned perpetrators of the genocide against the Tutsi versus a community sample of survivors. *J Trauma Stress.* 2012;25(4):446–53. 10.1002/jts.21728 22865747

[5] Rubanzana W Hedt-Gauthier BL Ntaganira J : Exposure to genocide and risk of suicide in Rwanda: a population-based case-control study. *J Epidemiol Community Health.* 2015;69(2):117–22. 10.1136/jech-2014-204307 25488977 PMC4316837

[6] Rieder H Elbert T : Rwanda - lasting imprints of a genocide: trauma, mental health and psychosocial conditions in survivors, former prisoners and their children. *Confl Health.* 2013;7(1):6. 10.1186/1752-1505-7-6 23531331 PMC3620568

[7] Munyandamutsa N Mahoro Nkubamugisha P Gex-Fabry M : Mental and physical health in Rwanda 14 years after the genocide. *Soc Psychiatry Psychiatr Epidemiol.* 2012;47(11):1753–61. 10.1007/s00127-012-0494-9 22402589

[8] Rugema L Mogren I Ntaganira J : Traumatic episodes experienced during the genocide period in Rwanda influence life circumstances in young men and women 17 years later. *BMC Public Health.* 2013;13:1235. 10.1186/1471-2458-13-1235 24373422 PMC3880849

[9] Perroud N Rutembesa E Paoloni-Giacobino A : The Tutsi genocide and transgenerational transmission of maternal stress: epigenetics and biology of the HPA axis. *World J Biol Psychiatry.* 2014;15(4):334–45. 10.3109/15622975.2013.866693 24690014

[10] Roth M Neuner F Elbert T : Transgenerational consequences of PTSD: risk factors for the mental health of children whose mothers have been exposed to the Rwandan genocide. *Int J Ment Health Syst.* 2014;8(1):12. 10.1186/1752-4458-8-12 24690436 PMC3978019

[11] Yehuda R Bell A Bierer LM : Maternal, not paternal, PTSD is related to increased risk for PTSD in offspring of Holocaust survivors. *J Psychiatr Res.* 2008;42(13):1104–11. 10.1016/j.jpsychires.2008.01.002 18281061 PMC2612639

[12] American Psychiatric Association, Task Force on DSM-IV: Diagnostic and statistical manual of mental disorders: DSM-IV.4th ed. Washington, DC: American Psychiatric Association;1994;xxvii:886. Reference Source

[13] Paul B Tollefsbol TO : Outline of Epigenetics.In Peedicayil J, Grayson DR, Avramopoulos D, editors. *Epigenetics in Psychiatry*. San Diego, CA: Elsevier;2014;27–44. 10.1016/B978-0-12-417114-5.00002-4

[14] Gapp K Soldado-Magraner S Alvarez-Sánchez M : Early life stress in fathers improves behavioural flexibility in their offspring. *Nat Commun.* 2014;5:5466. 10.1038/ncomms6466 25405779

[15] Gapp K Jawaid A Sarkies P : Implication of sperm RNAs in transgenerational inheritance of the effects of early trauma in mice. *Nat Neurosci.* 2014;17(5):667–9. 10.1038/nn.3695 24728267 PMC4333222

[16] Bale TL : Epigenetic and transgenerational reprogramming of brain development. *Nat Rev Neurosci.* 2015;16(6):332–44. 10.1038/nrn3818 25921815 PMC7064155

[17] Mulligan CJ D'Errico NC Stees J : Methylation changes at *NR3C1* in newborns associate with maternal prenatal stress exposure and newborn birth weight. *Epigenetics.* 2012;7(8):853–7. 10.4161/epi.21180 22810058 PMC3427280

[18] Yehuda R Daskalakis NP Bierer LM : Holocaust Exposure Induced Intergenerational Effects on *FKBP5* Methylation. *Biol Psychiatry.* 2016;80(5):372–80. 10.1016/j.biopsych.2015.08.005 26410355

[19] Kertes DA Kamin HS Hughes DA : Prenatal Maternal Stress Predicts Methylation of Genes Regulating the Hypothalamic-Pituitary-Adrenocortical System in Mothers and Newborns in the Democratic Republic of Congo. *Child Dev.* 2016;87(1):61–72. 10.1111/cdev.12487 26822443 PMC4733886

[20] Association AP : American Psychiatric Association’s Diagnostic and Statistical Manual of Mental Disorders (DSM-IV).1994;1232.

[21] Hothorn T Everitt B : A Handbook of Statistical Analyses Using R. Third ed. Boca Raton, FL: CRC Press;2014. 10.1201/b17081

[22] Penny W : Bayesian Inference for the Multivariate Normal.Wellcome Trust Center for Neuroimaging: University College, London.2014. Reference Source

[23] Shrira A Ayalon L Bensimon M : Parental Post-traumatic Stress Disorder Symptoms Are Related to Successful Aging in Offspring of Holocaust Survivors. *Front Psychol.* 2017;8:1099. 10.3389/fpsyg.2017.01099 28706503 PMC5489676

[24] Ainsworth AM : Constructing Rwandan Identity in the Diaspora: Remembering the Green Hills in Cold Canada.2015. Reference Source

[25] Bowers ME Yehuda R : Intergenerational effects of PTSD on offspring glucocorticoid receptor methylation. In *Epigenetics and Neuroendocrinology.* Springer, Cham.2016;141–155. 10.1007/978-3-319-29901-3_6

[26] Yehuda R Lehrner A : Intergenerational transmission of trauma effects: putative role of epigenetic mechanisms. *World Psychiatry.* 2018;17(3):243–257. 10.1002/wps.20568 30192087 PMC6127768

[27] Shrira A Mollov B Mudahogora C : Complex PTSD and intergenerational transmission of distress and resilience among Tutsi genocide survivors and their offspring: A preliminary report. *Psychiatry Res.* 2019;271:121–123. 10.1016/j.psychres.2018.11.040 30472506

[28] American Psychiatric Association: Diagnostic and Statistical Manual of Mental Disorders. Fifth Edition, Arlington, VA American Psychiatric Association,2013. Reference Source

[29] Hinton DE Lewis-Fernández R : The cross-cultural validity of posttraumatic stress disorder: implications for DSM-5. *Depress Anxiety.* 2011;28(9):783–801. 10.1002/da.20753 21910185

[30] Mutesa L : PTSD symptom domain data. OSF,2019. 10.17605/OSF.IO/P94TA

