# Supplementary material for: Transgenerational effects of the genocide against the Tutsi in Rwanda: A post-traumatic stress disorder symptom domain analysis
Source: AAS Open Res. 2020 Jan 14;1:10. Originally published 2018 Apr 18. [Version 2] doi: 10.12688/aasopenres.12848.2 (PMC11231627; doi:10.12688/aasopenres.12848.2)
Supplement: Peer review history

# Reviewer report: Reviewer response for version 2

**DOI:** 10.21956/aasopenres.14128.r31451

**Published:** 2024-07-08

**Authors:** Mirela Ambeskovic

**Affiliations:** 1 University of Lethbridge, Lethbridge, Alberta, Canada

The manuscript titled "Transgenerational effects of the genocide against the Tutsi in Rwanda: A post-traumatic stress disorder symptom domain analysis" is very novel and insightful. This is the first time such research has been performed on a human cohort in the sub-Sahara. The main outcomes of this article provide enough evidence to investigate further transgenerational stress in populations who were subjected to genocide. 

 Although this manuscript deficit in statistical strength (n=50) for a human study, it provides pilot data and conclusions that can be further explored.

Is the work clearly and accurately presented and does it cite the current literature?

Yes

If applicable, is the statistical analysis and its interpretation appropriate?

Partly

Are all the source data underlying the results available to ensure full reproducibility?

Yes

Is the study design appropriate and is the work technically sound?

Yes

Are the conclusions drawn adequately supported by the results?

Yes

Are sufficient details of methods and analysis provided to allow replication by others?

Partly

Reviewer Expertise:

Behavioural neuroscience with a focus on transgeenrational and ancestral stress.

I confirm that I have read this submission and believe that I have an appropriate level of expertise to confirm that it is of an acceptable scientific standard.

# Reviewer report: Reviewer response for version 2

**DOI:** 10.21956/aasopenres.14128.r27344

**Published:** 2020-02-13

**Authors:** Andreas Maercker

**Affiliations:** 1 Department of Psychology Psychopathology and Clinical Intervention, University of Zurich, Zurich, Switzerland

A plus of version 2 is the streamlined statistics. Its steps and significance estimates are now much easier to understand.

 Regarding the intensive analyses of the correlations of the symptom values with the PTSD total score, I can only state that this would not be done in the conventional psychometric tradition, because all correlations are above .85 - thus very high and with a ceiling effect.

 More important are the correlations with the epigenetic markers. I am not an expert on epigenetics. Despite the discussion of the authors, the associations seem to me to be quite low - but that's for others to decide.

Is the work clearly and accurately presented and does it cite the current literature?

Partly

If applicable, is the statistical analysis and its interpretation appropriate?

Partly

Are all the source data underlying the results available to ensure full reproducibility?

Partly

Is the study design appropriate and is the work technically sound?

Partly

Are the conclusions drawn adequately supported by the results?

Partly

Are sufficient details of methods and analysis provided to allow replication by others?

Partly

Reviewer Expertise:

NA

I confirm that I have read this submission and believe that I have an appropriate level of expertise to confirm that it is of an acceptable scientific standard.

# Reviewer report: Reviewer response for version 1

**DOI:** 10.21956/aasopenres.13914.r26485

**Published:** 2018-10-19

**Authors:** Andreas Maercker

**Affiliations:** 1 Department of Psychology Psychopathology and Clinical Intervention, University of Zurich, Zurich, Switzerland

Good to have this study. The sample(s) are very important and the general research questions are valueable.

 However, some aspects have to be changed before indexing:

1. Too few important statistical data are given. Reporting structural equation model results requires providing tables with its conventional statistical specifications, in the case of CFA: Chi-Squared test, RMSEA, GFI, AGFI, RMR, and SRMR
  The results of these indicators have to be evaluated, i.e. if they are in the sufficient range of values. Otherwise these results cannot be interpreted. Usually, most final CFA model comparisons seem sufficient but with some outliers that have to be interpreted.
2. Other data in the current text are highly repetitive, e.g. the Betas of the CFAs are reported threefold identically: in the text, in the figures, and the tables. This is against the rules of scientific publication which mandates to report all results only once.
  In the discussion the authors interpret their symptom group dominance results "intuitively" (p. 7, 1st paragraph). This is not good science. Why not cross-checking various PTSD models by authors such as Brewin *et al.*, Ehlers & Clark etc. why in one case recollections are most prominent and in others hyperarousal symptoms? Furthermore, why not reflecting on existing research literature that avoidance symptoms are usually the least prevalent in samples from Africa? (see: Hinton, D. E., & Lewis‐Fernández, R. (2011). The cross‐cultural validity of posttraumatic stress disorder: implications for DSM‐5. *Depression and anxiety*, *28*(9), 783-801)

Is the work clearly and accurately presented and does it cite the current literature?

Partly

If applicable, is the statistical analysis and its interpretation appropriate?

Partly

Are all the source data underlying the results available to ensure full reproducibility?

Partly

Is the study design appropriate and is the work technically sound?

Partly

Are the conclusions drawn adequately supported by the results?

Partly

Are sufficient details of methods and analysis provided to allow replication by others?

Partly

Reviewer Expertise:

NA

I confirm that I have read this submission and believe that I have an appropriate level of expertise to confirm that it is of an acceptable scientific standard, however I have significant reservations, as outlined above.

# Reviewer report: Reviewer response for version 1

**DOI:** 10.21956/aasopenres.13914.r26524

**Published:** 2018-08-28

**Authors:** Amit Shrira

**Affiliations:** 1 The Interdisciplinary Department of Social Sciences, Bar-Ilan University, Ramat-Gan, Israel

This paper examined the factor structure of PTSD syndromes among exposed and non-exposed mothers and offspring. I believe that the topic would be of interest to readers. Nevertheless, my primary concern regarding this paper is that the conclusions are based on descriptive statistics. It seems the authors did not examine measurement invariance across the study groups. Without such tests, it precludes the interpretation of their findings. The reader is left wondering whether the coefficient for intrusive recollection is indeed significantly different across groups or relative to other coefficients.

 Moreover, it is not clear if the covariances between PTSD syndrome and methylation are significant or not. I also wondered why the authors used group means DNA methylation values rather than individual DNA methylation. If that information was not available, the authors should explain what was the reason for this secondary analysis approach.

 Finally, the paper can benefit by relating the current findings with previous ones. For example, the paper by Bowers and Yehuda (Bowers, M. E., & Yehuda, R. (2016). Intergenerational effects of PTSD on offspring glucocorticoid receptor methylation. In D., Spengler, & E. Binder (Eds.), *Epigenetics and neuroendocrinology: Clinical focus on psychiatry *(vol. 2, pp. 141-155). Switzerland: Springer) reviews additional studies looking at the methylation in NR3C1. Shrira and colleagues (Shrira, A., Ayalon, L., Bensimon, M., Bodner, E., Rosenbloom, T., & Yadid, Y. (2017). Parental posttraumatic stress disorder symptoms is related to successful aging in offspring of Holocaust survivors. *Frontiers in Psychology, 8*. 1099) suggest such methylation patterns can explain greater mental as well as physical morbidity across generations in exposed families.

Is the work clearly and accurately presented and does it cite the current literature?

Partly

If applicable, is the statistical analysis and its interpretation appropriate?

No

Are all the source data underlying the results available to ensure full reproducibility?

Partly

Is the study design appropriate and is the work technically sound?

Partly

Are the conclusions drawn adequately supported by the results?

No

Are sufficient details of methods and analysis provided to allow replication by others?

Partly

Reviewer Expertise:

NA

I confirm that I have read this submission and believe that I have an appropriate level of expertise to confirm that it is of an acceptable scientific standard, however I have significant reservations, as outlined above.
